# A current view of serotonin transporters

**DOI:** 10.12688/f1000research.8384.1

**Published:** 2016-07-28

**Authors:** Louis J. De Felice

**Affiliations:** 1 Physiology and Biophysics, School of Medicine, Virginia Commonwealth University, Richmond, VA, USA

**Keywords:** 5-HT, alternating access model, SLC6, electrogenic transport, Serotonin transporters

## Abstract

Serotonin transporters (SERTs) are largely recognized for one aspect of their function—to transport serotonin back into the presynaptic terminal after its release. Another aspect of their function, however, may be to generate currents large enough to have physiological consequences. The standard model for electrogenic transport is the alternating access model, in which serotonin is transported with a fixed ratio of co-transported ions resulting in net charge per cycle. The alternating access model, however, cannot account for all the observed currents through SERT or other monoamine transporters.  Furthermore, SERT agonists like ecstasy or antagonists like fluoxetine generate or suppress currents that the standard model cannot support.  Here we survey evidence for a channel mode of transport in which transmitters and ions move through a pore. Available structures for dopamine and serotonin transporters, however, provide no evidence for a pore conformation, raising questions of whether the proposed channel mode actually exists or whether the structural data are perhaps missing a transient open state.

What happens when 5-hydroxytryptamine (5-HT) and therapeutic or abused drugs interact with the serotonin transporter? In addition to pharmacokinetics, drug metabolism, and the complexities of the serotonergic system, the events occurring directly at the serotonin transporter (SERT) will help us understand how therapeutic and abused drugs work. Neurotransmitter transporters and their role in synaptic transmission are broad subjects, and many topics, including the important role of 5-HT in the enteric nervous system, are not covered here. Previous reviews include ^1– 10^. This commentary focuses on the transporter and small molecules (neurotransmitters and drugs) that interact with the transporter. Our discussion inevitably involves the function of SERT in heterologous expression systems, other transporters like the dopamine transporter (DAT), and currently available structural data on the solute carrier 6 (SLC6) family of neurotransmitter transporters, of which both SERT and DAT are members. This commentary also broaches the concept of channel activity in SERT, as this property is one of the most controversial but least well understood.

Biochemical characterization of SERT began in 1977, when Gary Rudnick used plasma membrane vesicles from human platelets containing human SERT (hSERT) ^11^ to study Na ^+^ and Cl ^−^ coupled 5-HT transport. Rudnick *et al.* concluded that SERT, in common with NET (norepinephrine transporter) and DAT, has an obligatory functional dependence on Na ^+^ and Cl ^−^: 5-HT transport cannot occur without the co-transport of one Na ^+^ and one Cl ^−^ ion. The standard model for monoamine transporters, and SERT in particular, thus posits fixed stoichiometry of transmitter and ions, which, in the presence of ion gradients, can drive a transmitter against its own gradient ^12– 22^. In one form of this model, the influx of 5-HT (a monovalent cation at physiological pH) with Na ^+^ and Cl ^−^ is coupled to the efflux of one K ^+^ ion, thus rendering SERT electroneutral ^23^. However, we now know that SERT is electrogenic and that 5-HT transport generates current. It is critical to understand the molecular basis of these currents because they figure prominently in the action of antidepressants and drugs of abuse ^22, 24– 29^.

Two models have been proposed to explain the intriguing phenomenon of ion currents, neither of which is mutually exclusive. One model incorporates both uptake and current in one kinetic scheme ^30^, and so in this sense these two events are coupled. Another model attributes current to fixed stoichiometry, electrogenic alternating access transport with the ‘uncoupled’ current perhaps being caused by an occasional ‘slip’ into a channel mode. Such a phenomenon may occur in *Drosophila* SERT (dSERT) ^31^, which exhibits remarkably high currents, with 50 charges per 5-HT (at −80 mV) versus 5–12 charges for its mammalian counterparts ^32^. In dSERT, one of the putative extracellular gating residues is a polar asparagine instead of an acidic glutamate, as in hSERT, and thus presumably would be able to form only a hydrogen bond rather than a stronger salt bridge with its positively charged (arginine) gating partner. Regardless of the model invoked to explain transporter currents, the role of these currents at the serotonergic synapse remains unclear.

An important experiment potentially related to this question was performed by Dieter Bruns *et al.*, who used an *in vivo* preparation: the giant serotonergic synapse of the medicinal leech ^33^. This work measured pre- and postsynaptic currents in response to timed presynaptic 5-HT release. Distinguishing between presynaptic versus postsynaptic events as well as transporter versus receptor current was achieved by specific, targeted placement of recording electrodes and well-established pharmacological intervention. Interestingly, the postsynaptic ionotropic 5-HT3 receptor generated current *after* the presynaptic current associated with 5-HT transport back into the presynaptic terminal. Only a brief delay occurred between a presynaptic Ca ^++^ flash, release of 5-HT, and a presynaptic SERT current that was earlier than, but comparable in size to, the post-synaptic, 5-HT3-gated receptor current. It would seem impossible for traditional models to explain such large SERT currents, and these data suggest that a channel may exist within SERT, although no structural evidence yet exists for such a channel.

A recent study ^34^ provided the first structural glimpse into hSERT, but only structures of a transport-deficient variant, dubbed “TS3”, in complex with two inhibitors, are described. The resolution of the transport-competent variant, dubbed “TS2”, is low (~4.5 Å) with no 5-HT, inhibitor or ions present in the electron density and consequently none in the deposited coordinate file. Higher-resolution structural studies coupled with sophisticated biophysical experiments of a transport-competent SERT in multiple conformations will be required to visualize a channel if one exists. Nevertheless, such a channel-like state may be transient and thus still be challenging to capture unless stabilized in some way. Note that existing crystal structures of homologous transporters, such as the bacterial SLC6 orthologue LeuT, which is widely used to model plasma membrane monoamine transporters, along with recent structures of *Drosophila* DAT (dDAT), provide evidence for the fixed stoichiometry, alternating access model, with no hint of a channel ^35– 38^. However, the absence of such a conformation does not disprove its existence. Indeed, the proteins used for crystallization either do not exhibit channel activity (LeuT) or are highly thermostabilized, transport-deficient/impaired mutants (hSERT and dDAT) that may simply be incapable of sampling the channel-like state present in the wild-type proteins.

Unlike the biogenic amine transporters, glutamate transporters, which belong to a functionally and structurally distinct neurotransmitter transporter family (SLC1), have been known for decades to embody an authentic Cl ^−^ channel and thus operate normally as both a glutamate carrier and a Cl ^−^ ion channel ^39– 45^. Is there any structural evidence for a channel in this family? Structural breakthroughs within the SLC1 family have emerged from work on an archaeal glutamate transporter homolog from *Pyroccocus horikoshii*, known as Glt _ph_^46^. Importantly, Glt _ph_ has a bona-fide Cl ^−^ channel ^47^, akin to its eukaryotic counterparts. In the crystal structure of a crosslinked Glt _ph_ mutant (Glt _ph_-V198C-A380C) with bound mercury, one of the protomers of the asymmetric trimer serendipitously assumes an intermediate outward-facing state in which a small cavity between the transport and trimerization domains appears to be accessible from both sides of the membrane. Although this conformation may be stabilized by crystal packing, as the authors remark, the structure offers a tantalizing molecular glimpse into a plausible channel-like state ^48^. By contrast, as mentioned above, none of the SLC6 structures solved to date reveals such an open pathway.

If a channel exists within SLC6 members, do currents that carry Na ^+^ or Cl ^−^ ions share a pathway with 5-HT? In 2011, Henry *et al.* studied the effect of an asparagine-to-alanine mutation at position 101 in TM1 of hSERT ^49^, which, according to LeuT-based homology models and the recent hSERT structure, resides within the transport permeation pathway, close to the putative 5-HT and ion-binding sites. Henry *et al.* found that the N101A mutation eliminated Cl ^−^ dependence of 5-HT transport, 5-HT-induced currents, and 5-HT-independent leak currents. In fact, the N101A mutant evoked a much greater 5-HT/charge flux ratio (relative to that found in the wild-type) despite its reduced surface expression. These data suggest that N101 likely determines both Na ^+^-coupled transport and channel pathways.

We also know that SERT and DAT have distinct functional states ^50– 52^, and although these may reflect distinct structural pathways, there is no proof of this. However, we must conclude that perfectly coupled, fixed stoichiometric models are incomplete, if for no other reason than the existence of leak currents ^53^. Furthermore, 5-HT-induced macroscopic currents are 100 to 1,000 times larger than can be explained by a fixed stoichiometry, alternating access model, and this is another indirect argument for the existence of a channel-like pathway in SERT ^4^.

Theories of transport based on flux coupling (also called frictional models) can account for observed macroscopic currents and are consistent with the existence of single transporter events ^4, 31, 33, 54– 59^. Then how is it possible that present structural data of plasma membrane monoamine transporters provide no evidence for the existence of current-generating ion pathways? Perhaps channel states in this class of transporters are unstable, simply resistant to structural analysis, especially (as mentioned above) with the thermostabilized constructs normally required to grow diffraction-quality crystals, or perhaps they need to be crystallized within a membrane-like environment, as would occur in the lipidic cubic phase ^60^ of phospholipid bilayer nanodiscs. Perhaps both a current-generating construct combined with a lipidic milieu are necessary and, though entirely speculative, may need to be partnered with a presynaptic regulatory protein as would actually occur in the cell. Regardless, without structural evidence for channels in plasma membrane monoamine transporters, the existing functional data will lie dormant.

Let us return to the original question: What happens when 5-HT, therapeutic drugs, or abused drugs arrive at the serotonin transporter? As 5-HT is transported, SERT generates large depolarizing currents that are incompatible with fixed stoichiometry models but consistent with channel models ^4, 27, 31, 61^. For drugs of abuse, consider as an example that ecstasy generates larger currents through SERT than 5-HT does. The resulting depolarization due to these currents is sufficient to open voltage-gated Ca ^++^ channels ^29^. The influx of Ca ^++^ could itself have effects, but certainly the resulting depolarization in serotonergic neurons would increase excitability and presynaptic 5-HT release. On the other hand, when uptake inhibitors interact with hSERT, in addition to blocking uptake they also block the inherent transporter leak current and that would hyperpolarize serotonergic neurons and decrease the probability of 5-HT release. These few examples suggest a larger picture of how transporter agonists and antagonists may influence the function of SERT in neurons given the channel-like properties of SERT. In conclusion, SERT is not only a 5-HT carrier but also may transiently act as an ion channel poised for pharmacological manipulation.
